# Pulsatile flow dynamics maintain pulmonary artery architecture

**DOI:** 10.1172/jci.insight.201797

**Published:** 2026-05-08

**Authors:** Stephen B. Spurgin, Lauren Thai, Tina C. Wan, Christopher P. Chaney, Mitzy A. Cowdin, Surendranath Veeram Reddy, Tarique Hussain, Munes Fares, M. Luisa Iruela-Arispe, Thomas Carroll, Andrew D. Spearman, Ondine Cleaver

**Affiliations:** 1 Department of Molecular Biology, and; 2 Heart Center, Children’s Health, Division of Cardiology, Department of Pediatrics, UT Southwestern Medical Center, Dallas, TX, USA.; 3 Department of Pediatrics, Division of Cardiology, Medical College of Wisconsin, Children’s Wisconsin, Herma Heart Institute, Milwaukee, Wisconsin, USA.; 4 Cardiovascular Center, Medical College of Wisconsin, Milwaukee, Wisconsin, USA.; 5 Department of Internal Medicine and Division of Nephrology, and; 6 Department of Radiology, University of Texas Southwestern Medical Center, Dallas, Texas, USA.; 7 Department of Cell and Developmental Biology, Northwestern University Feinberg School of Medicine, Chicago, Illinois, USA.

**Keywords:** Cardiology, Vascular biology, Cardiovascular disease, Endothelial cells, Growth factors

## Abstract

Single-ventricle congenital heart disease (SV-CHD) is a uniformly lethal condition requiring the Glenn surgery, which as a side effect eliminates arterial pulsatility and contributes to pulmonary vascular complications. In Glenn patients, we quantified pulsatility loss in each dimension of force (flow, pressure, and stretch) using cardiac catheterization and MRI. To model and investigate the individual impact of each dimension of pulsatility loss on the pulmonary vasculature, we applied isolated pulsatile and non-pulsatile mechanical stimuli to pulmonary artery endothelial cells (ECs) in vitro. We found that each dimension of force triggered distinct transcriptional responses, revealing force-specific regulation of structural and signaling pathways. Pulsatile stretch uniquely stimulated EC secretion of PDGFB, a key driver of vascular smooth muscle cell (vSMC) recruitment. In a rat Glenn model, loss of pulsatility led to vascular wall thinning, loss of EC PDGFB, and reduced activation of smooth muscle PDGFBRβ, confirming in vivo relevance. Our findings uncover a mechanistic link between endothelial stretch sensing and PDGFB-mediated EC-vSMC crosstalk, essential for maintaining pulmonary artery architecture. Clinically, these insights suggest that restoring or mimicking pulsatile forces may help preserve vascular integrity and prevent remodeling in patients with SV-CHD.

## Graphical abstract

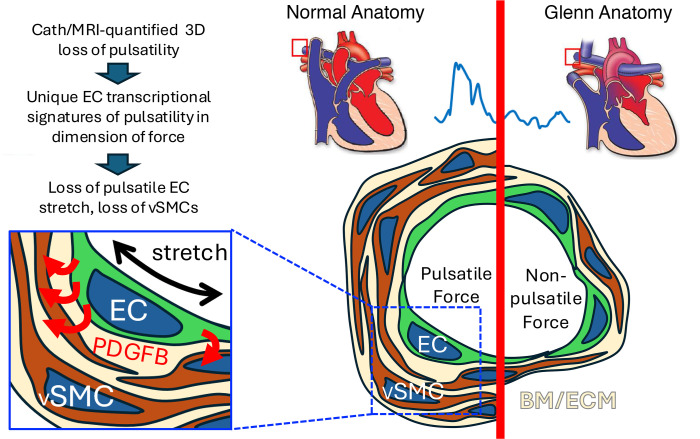

## Introduction

Globally, 1 in every 2000 babies is born with a heart that has a single functional ventricle (1). Surviving this congenital abnormality requires early and significant medical intervention. A single ventricle cannot perform the work of two for very long, nor can it direct an equal amount of blood flow to the lungs (low vascular resistance) and the body (high vascular resistance). For children with single-ventricle congenital heart disease, survival depends on a series of remarkable surgeries: the Glenn and the Fontan. In the circulation created by these procedures, venous blood flows directly to the lungs without the support of a pumping ventricle, while the single functional ventricle supplies pulsatile flow to the rest of the body (2).

A frequent consequence of surgical replumbing of the vasculature in single-ventricle patients is the development of a wide variety of vascular malformations: (a) diffuse microvascular pulmonary arteriovenous malformations (PAVMs) in the Glenn stage and (b) larger, tortuous collateral vessels in both Glenn and Fontan stages. It has been shown that the microvascular lesions that arise in the Glenn are reversible with restoration of the direct flow of liver blood to the lungs in the Fontan (even though they still lack cardiac pulsatile flow). This has led to the “hepatic factor” hypothesis (3–5) whereby a liver factor lacking in the Glenn may be the critical element. By contrast, the proximal macrovascular collateral vessels do not regress in the Fontan (6, 7). Among the abundant potential pathogenic events in these models, including chronic hypoxemia, chronic inflammation, and loss of direct hepatopulmonary blood flow (3, 5, 8), a role for pulsatility has long been suggested (9–11). However, this possibility has never been investigated at a mechanistic level.

Pulsatility, for its part, is a critical characteristic of arterial blood flow. Arteries — whether systemic, pulmonary, or umbilical — are defined by the presence of pulsatile blood flow, not by their color or oxygen content. The structure of the arterial wall, which includes thick smooth muscle layer and elastic laminae, reflects the need to withstand the high pulsations of arterial pressure and stretch. Veins, on the other hand, have less hemodynamic pulsatility and a thinner smooth muscle layer (media) (12). Critically, the histopathology of vascular malformations (VMs) often reveals absent, patchy coverage of the smooth muscle layer (13, 14) and thinning of the vascular wall (15). During VM formation, this mural insulation breaks down, allowing formation of new, aberrant connections between vessels. Increased endothelial cell (EC) proliferation then contributes to the pathogenesis of VMs (13). Once a connection is formed, the transmission of pulsatile, arterial blood flow to venous vessels induces inappropriate “arterialization” (medial thickening, intimal hyperplasia) of the venous vessels (16, 17), further highlighting the relationship between arterial hemodynamics and the architecture of the vascular wall.

The impact of cardiac pulsatility on the arterial side of the vasculature has been noted from the early stages of vascularization (18). However, the role of pulsatility in maintaining arterial and venous endothelial identity and function after birth has received very limited attention. Scientific investigation of EC mechanobiology to date has focused on other physical forces, such as the cellular impact of low versus high shear stress, or of laminar versus disturbed flow (19). Despite the clinical centrality of pulsatility, it remains an under-investigated aspect of vascular biology.

ECs are remarkably sensitive to the physical forces transmitted by blood flow. Endothelial mechanosensitive components include cell-cell adhesion molecules (integrins, gap junctions, cadherins), extracellular matrix–sensing (ECM-sensing) components, ion channels, and more (19–21). A mechanosensor located at EC-EC junctions has been shown to act as a molecular strain gauge, converting the physical forces of blood flow into biochemical signals that regulate vascular tone and remodeling (21). The forces sensed by these molecular components are applied to ECs in 3 dimensions: shear stress, pressure, and stretch (22). Each dimension of force therefore exerts distinct stresses and deformations to ECs. Laminar shear stress (LSS) is the frictional drag force that occurs along the inner (apical) surface of the vessel endothelium (23). Stretch is the effect downstream of each heartbeat, as vessels widen radially to accommodate an ejected volume of blood and their circumference expands (24). Hydrostatic pressure is generated by ventricular contraction, and the driving force behind stretch (25). Notably, ECs align parallel to the direction of LSS (blood flow) but perpendicular to the direction of stretch (26), further highlighting the need to consider each dimension’s impact separately. All 3 biomechanical forces have been shown to alter vascular ECM (27), as well as endothelial and smooth muscle transcriptional programs (28, 29). However, the unique impact of pulsatility within each dimension of force on vascular homeostasis remains a critical knowledge gap.

The endothelium and vascular smooth muscle communicate closely. Critically, crosstalk between ECs and vascular smooth muscle cells (vSMCs) is mediated by EC secretion of platelet-derived growth factor B (PDGFB), which binds to PDGF receptor β (PDGFRβ) on vSMCs, driving proliferation and promoting mural cell coverage in large vessels down to the capillary level (30–32). Loss of Notch signaling in pericytes downregulates *Pdgfrb* in vSMCs in mouse models, leading to AVMs (33). Deleting PDGFB specifically from ECs results in remarkable dilation and arteriovenous shunting in the lungs of mice (34), and loss of PDGFB has been observed in human VMs (35). Conversely, in patients with pulmonary hypertension — with pathologic pulmonary vSMC growth — there is increased PDGFB and PDGFRβ in the ECs and vSMCs, respectively (36), and treatment with PDGFRβ blockade can alleviate their symptoms (37). The question arises as to whether different hemodynamic forces coordinate the maintenance of vascular structure via EC responses to these forces.

Here, we investigate the biomechanical forces experienced by pulmonary artery (PA) ECs in Glenn patients and identify pulsatility of blood flow as critical to maintenance of the lung vasculature. We first use combined cardiac catheterization (cath) and cardiac magnetic resonance imaging (MRI) to quantify and compare the pulsatility of force that is applied to PA ECs in children with normal cardiopulmonary anatomy and in those with Glenn circulation. After noting a clear loss of pulsatility in the Glenn PAs, we model those changes using cultured human PA ECs (HPAECs) to define the specific transcriptional programs driven by each individual dimension of force. We demonstrate that pulsatile stretch in vitro, which mimics expansion of the arterial vessel intima during cardiac ventricular systole, significantly impacts the transcriptional landscape of ECs. Specifically, we show that pulsatile stretch stimulates ECs to secrete PDGFB. In addition, using our previously published in vivo rat model of the Glenn surgery, where PAs experience loss of pulsatile stretch, we demonstrate significant reduction of PDGFB in PA ECs. Finally, we build on our recent work in a rat model of the Glenn surgery (38), which showed early and progressive arteriovenous shunting, to demonstrate significant thinning of PA vascular walls. We show both loss of EC-derived PDGFB expression and loss of p-PDGFRβ in vSMCs. Our study identifies pulsatile stretch as a critical and underappreciated dimension of arterial force that promotes vascular smooth muscle endowment, thereby promoting arterial structural support. Gaining insight into the impact of pulsatility of hemodynamic force is a critical approach to understanding clinically relevant EC biology.

## Results

### The Glenn shunt directs venous blood flow to PAs.

**Figure 1:**
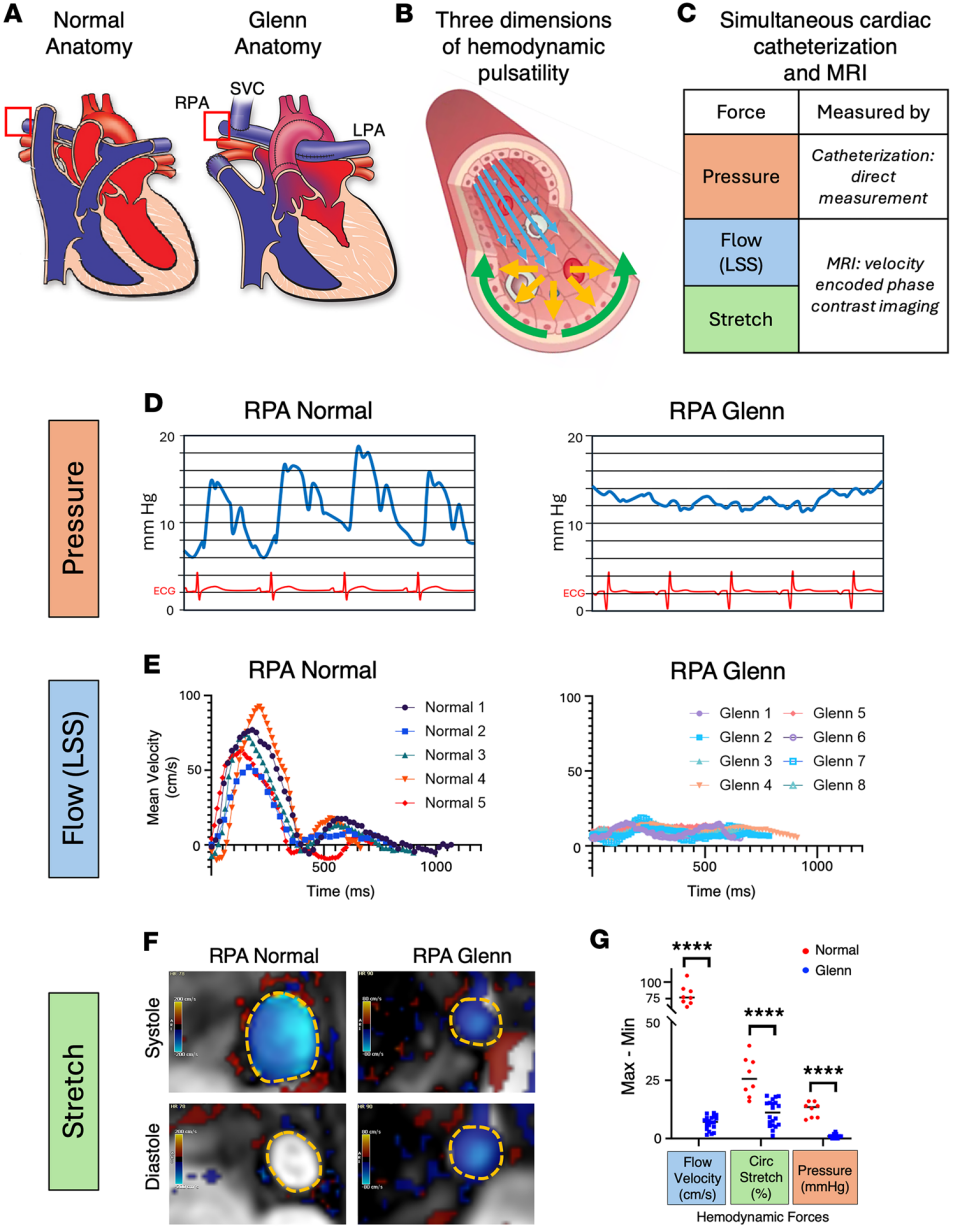
Loss of pulsatility in 3 dimensions within Glenn pulmonary arteries. (**A**) Diagrams of representative normal and Glenn cardiopulmonary anatomy. Red box highlights location of force pulsatility measurements at the right pulmonary artery (RPA). LPA, left pulmonary artery; SVC, superior vena cava. (**B**) Hemodynamic forces act on ECs in 3 dimensions: laminar shear stress (LSS)/flow (blue), pressure (orange), and circumferential stretch (green). Figure created in Biorender. (**C**) Outline of data collection method for each dimension of hemodynamic force. (**D**) Example pressure waveforms from the RPA of normal and Glenn patients. Electrocardiogram shown in red for correlation to cardiac cycle. (**E**) Variation in flow velocity of the proximal RPA measured by cardiac MRI throughout a cardiac cycle from systole (start) to end-diastole (end). Variability in length of time for each patient reflects the variable heart rate within each participant. (**F**) Cardiac MRI velocity–encoded phase-contrast images, with color showing velocity of blood flow out of the plane of the image slice. Dotted orange line shows RPA border. RPA diameter increases during systole in a normal patient but does not increase during systole in Glenn patient. (**G**) Quantification of pulse difference of each dimension of force, obtained during combined cardiac catheterization and cardiac MRI of normal (*n* = 20) and Glenn (*n* = 20) patients, demonstrating loss of pulsatility in the Glenn. Circumference change (%) was calculated from baseline in diastole. ****P ≤ 0.0001 by unpaired, 2 tailed Student’s *t* test with Welch’s correction.

Arterial vessels normally experience blood flow directly from a pumping cardiac ventricle, adding pulsatility to the magnitude of hemodynamic forces (Figure 1A). By reviewing the known characteristics of 3 distinct pairings of arteries and veins (systemic, pulmonary, and umbilical) in human, we note that pulsatile flow is a shared, definitional quality of arteries (Supplemental Figure 1; supplemental material available online with this article; https://doi.org/10.1172/jci.insight.201797DS1). Pulsatility is thus an inherent quality of blood flow in arterial vessels. After a Glenn surgery, however, the PAs no longer receive blood flow directly from a pumping heart ventricle. Instead, pulmonary blood flow is provided by the Glenn shunt: direct venous flow through the superior vena cava (SVC), which is anastomosed to the PAs (Figure 1A). ECs that make up the inner lining of PAs downstream of normal cardiac vessel anatomy are subjected to force in 3 dimensions: flow/sheer stress, pressure, and circumferential stretch (Figure 1B) (22). Here, we assess these forces in the Glenn lung arteries.

### Pulsatility loss in 3 dimensions in Glenn patients.

To quantitatively determine the loss of PA pulsatility in patients with Glenn anatomy, we used combined cardiac catheterization and cardiac MRI (cath/MRI) in 20 Glenn patients to obtain simultaneous data in all 3 dimensions of force: pressure, flow, and circumferential stretch (Figure 1, B and C). These data were compared to data obtained in 20 age- and sex-matched patients with normal cardiopulmonary connections (see patient demographics in Supplemental Table 1). First, as has long been observed clinically, we observed pulsatility loss in the PA pressure of Glenn patients (Figure 1D). Similarly, we noted that the Glenn shunt leads to non-pulsatile flow (LSS) in the PAs, as seen by a comparison of the PA velocity profiles in normal and Glenn (Figure 1E) patients throughout the cardiac cycle. Finally, we found that the circumference of the proximal right PA (RPA) does not expand repeatedly with each heartbeat in Glenn patients, demonstrating loss of pulsatile circumferential stretch (Figure 1, F). Thus pulsatility is lost in all three dimensions of force in patients with the Glenn circulation (Figure 1G).

### In vitro modeling reveals unique transcriptome for each dimension of pulsatile force.

ECs are highly responsive to mechanical force, and can react to differences in both magnitude and direction of force (39). Given the observed loss of pulsatility in Glenn patients, we next investigated the relative impact of pulsatility within each dimension of force on ECs. Using our patient cath/MRI pulsatility data, we designed in vitro experiments that model the pulsatility loss defined in Glenn patients.

**Figure 2:**
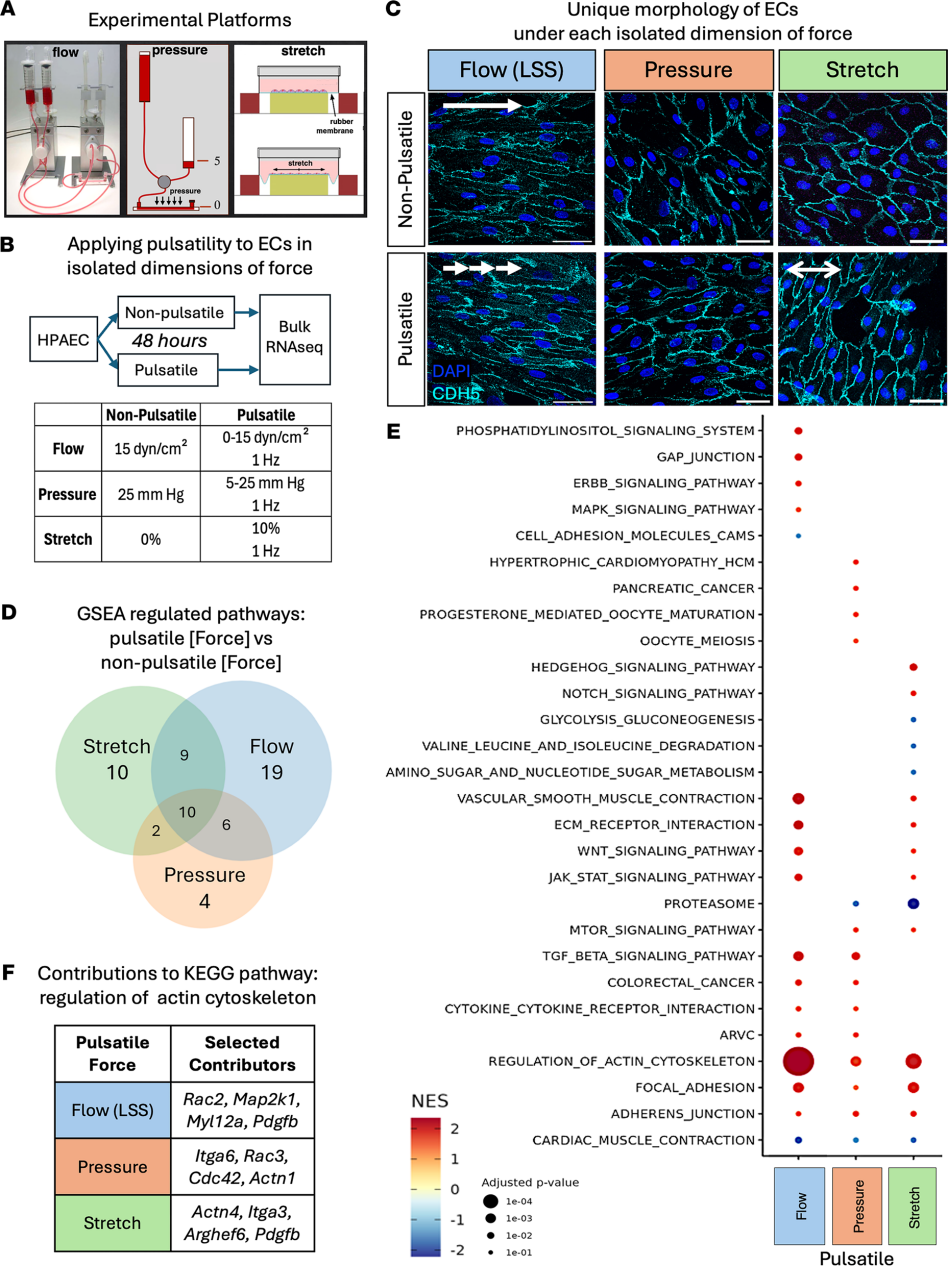
Pulsatility drives distinct signaling pathways within each dimension of force. (**A**) Diagram of cell culture setup to apply isolated dimensions of force to ECs. (**B**) Experimental conditions: HPAECs were exposed to 48 hours of a single dimension of either non-pulsatile or pulsatile force, and bulk RNA-seq was performed. (**C**) Representative images of HPAECs under flow (LSS), pressure, or stretch, displaying distinct cell-shape adjustments that occur in response to different forces, as revealed by immunofluorescent staining for the junction marker CDH5. Scale bar: 25μm. (**D**) GSEA of bulk RNA-seq data shows overlapping and unique pathways activated by each dimension of force. (**E**) Normalized enrichment score (NES) and adjusted *P* value shown for select signaling pathways shows greater impact of pulsatility within flow and stretch relative to pulsatility of pressure. (**F**) Key genes within the actin cytoskeleton pathway that are upregulated by each dimension of force.

To test the impact of each dimension of force on ECs, we exposed primary HPAECs to LSS, pressure, or stretch, using different flow platforms (Figure 2A). Each isolated, single dimension of force was applied to cultured HPAECs in a pulsatile and non-pulsatile manner. For pulsatile LSS, we applied 0–15 dyn/cm^2^ shear stress at 1 Hz, which mimics the frequency of a resting heart rate. For pulsatile pressure, we modified the Ibidi equipment to expose HPAECs to columns of media positioned to alternately provide 5 or 25 mmHg pressure, at 1 Hz, in the absence of flow or stretch. For stretch, we used a Flexcell system to stretch HPAECs by 10% of their baseline along a single axis (again at a frequency of 1 Hz). To assess stable transcriptional changes between non-pulsatile and pulsatile conditions, and thus improve in vivo relevance of our findings, HPAECs were exposed to 48 hours of force or pulsatile force (Figure 2B). To best mimic in vivo conditions in the years of the Glenn stage, pulsatile stretch was compared to no stretch rather than continuous stretch, which does not happen in vivo as it would lead to progressive dilation of the vessel. Importantly, we did not find significant changes in baseline expression of common EC genes between the 3 experimental setups (Supplemental Figure 2), indicating no basic alteration of endothelial fate. We observed similar physical changes to those we and others have previously reported (40, 41); ECs align parallel to the axis of flow and perpendicular to the axis of stretch, and exhibit no alignment preference under continuous or pulsatile pressure (Figure 2C). The unique, force-dependent changes in cell shape in response to each axis of force highlight the need to examine them individually.

We next carried out bulk RNA sequencing (RNA-seq) on HPAECs under each dimension of force, in a pulsatile or non-pulsatile manner (Supplemental Figure 5). We first investigated the overall pathways that were regulated by pulsatility. Using gene set enrichment analysis (GSEA), we noted that each dimension of force drove changes in unique signaling pathways, and that the impact of LSS was the most distinct. Overall, in HPAECs, laminar flow affected the greatest number of unique pathways (19), followed by stretch (10) and pressure (4) (Figure 2D). A wide range of pathway types were altered by biomechanical forces, many related to changes in structural cellular components, growth, or interaction of the cell with ECM (Figure 2E). We note that mitogen-activated protein kinase (*Mapk*) and epidermal growth factor receptor (*ErbB*, also known as *HER*) signaling were only induced by pulsatility of flow, while Hedgehog and Notch signaling were only induced by pulsatility of stretch. Pulsatile pressure did not uniquely regulate any obviously EC-related pathways but did upregulate the hypertrophic cardiomyopathy pathway.

In addition to these dimension-specific pathways, we found other pathways that are influenced across multiple dimensions of force. We found 10 pathways upregulated by all 3 dimensions of force, and 17 pathways induced by 2 of the 3 dimensions. We note that shear stress, stretch, and pressure all drove changes in the pathway “Regulation of Actin Cytoskeleton.” However, an analysis of the leading-edge subset (the genes within the pathway that are most significantly changed and thus responsible for the overall significance of the pathway) revealed upregulation of different genes by each dimension of force. For example, within the “Regulation of Actin Cytoskeleton” pathway, pulsatile pressure shows *Actn1* and pulsatile flow yields *Map2k1*, while pulsatile stretch promotes *Pdgfb*, *Arghef6*, and others (Figure 2F). Different dimensions of force therefore drive different transcriptional changes that converge on similar pathways.

### Stretch drives the majority of pulsatility-induced changes in PA EC gene expression.

**Figure 3:**
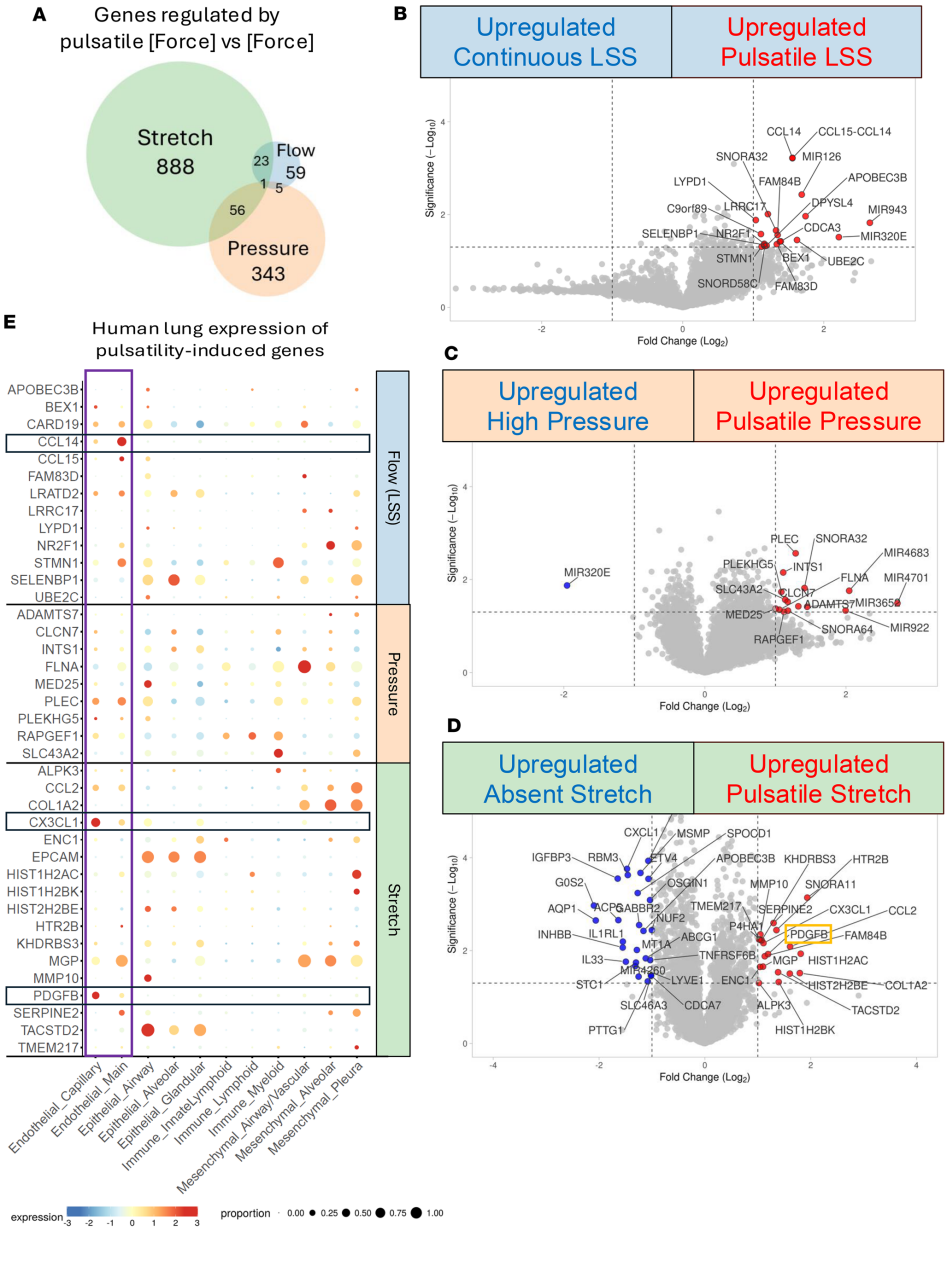
Transcriptional impact of pulsatility is greatest in the dimension of stretch, where it drives expression of the EC-specific gene *Pdgfb*. (**A**) Venn diagram of the number of genes significantly up- or downregulated by pulsatility of flow, pressure, or stretch (regardless of fold change). (**B**–**D**) Volcano plots of significantly regulated genes within each dimension of pulsatile force: (**B**) LSS, (**C**) pressure, and (**D**) stretch. (**E**) Genes upregulated by unique dimensions of pulsatile force in ECs were analyzed within the LungMAP single-cell RNA-seq data of human lung tissue (347,970 cells). Expression within ECs (purple box) was compared to other cell types. CCL14, CX3CL1, and PDGFB (black boxes) were uniquely expressed by ECs.

We next compared the differentially expressed genes induced by each dimension of force under pulsatile conditions. Strikingly, we found very little overlap between individual genes enriched in ECs under different dimensions of force, demonstrating unique responses to distinct forces. Of the genes significantly regulated by pulsatility, 93.4% (1205/1290) were driven by a single, unique dimension of force. Pulsatility of stretch accounted for over two-thirds (888 genes, 68.9%) of these genes (Figure 3A). Only 59 genes were uniquely induced by pulsatility of laminar flow, while 343 were uniquely induced by pulsatility of pressure. We found less overlap in individual genes regulated by pulsatility than there was in overall pathways. Twenty-three genes were regulated by pulsatility of both LSS and stretch, 56 genes by stretch and pressure, and 5 by pressure and LSS. Only a single gene (*Fam84B* or *Lratd2*) was upregulated by pulsatility in 3 dimensions of force.

We initially hypothesized that application of pulsatile force on cultured ECs would induce greater expression of genes previously shown to be specific to arteries (42). We asked whether the classical markers of arteriovenous EC differentiation might be promoted by pulsatile forces. Contrary to our predictions, we found no dimension of pulsatility that significantly affected the expression of most developmental arterial (*Efnb2*, *Eng*, *Sox17*, *Notch1*, *Jag1*, *Nrp1*, and *Bmx*) or venous (*Ephb4*, *Emcn*, *Nr2f1*, *Nr2f2*/*Coup-TFII*, and *Clu*) driver genes (Supplemental Figure 3). Similarly, we found no significant changes arterial or venous standard marker genes in Glenn rate arterial ECs compared to sham operated rats (data not shown).

We next asked whether there were genes specifically induced by pulsatile LSS, pressure, or stretch, when compared with the non-pulsative forces. After applying a *P*-value cutoff of 0.05 and fold-change cutoff of 2, we found that pulsatile stretch drove a change in 44 genes, compared with 20 genes by pulsatility of flow, and 16 genes by pulsatility of pressure (Figure 3, B–D). Expression of these hits in data from other dimensions of force is shown in Supplemental Figure 4. Genes upregulated under pulsatile LSS included many factors involved in endothelial activation, inflammation, and proliferation (Figure 3B), while genes upregulated under pulsatile pressure included factors associated with cytoskeletal remodeling, cell adhesion, and RNA processing (Figure 3C). Gene expression was particularly impacted by application of pulsatile stretch, with many factors involved in tissue remodeling, inflammatory activation, and cellular stress responses being upregulated (Figure 3D), including factors driving cell division and cell survival pathways (Figure 3D).

### Identification of Pdgfb as a stretch-induced gene in PA endothelium.

**Figure 4:**
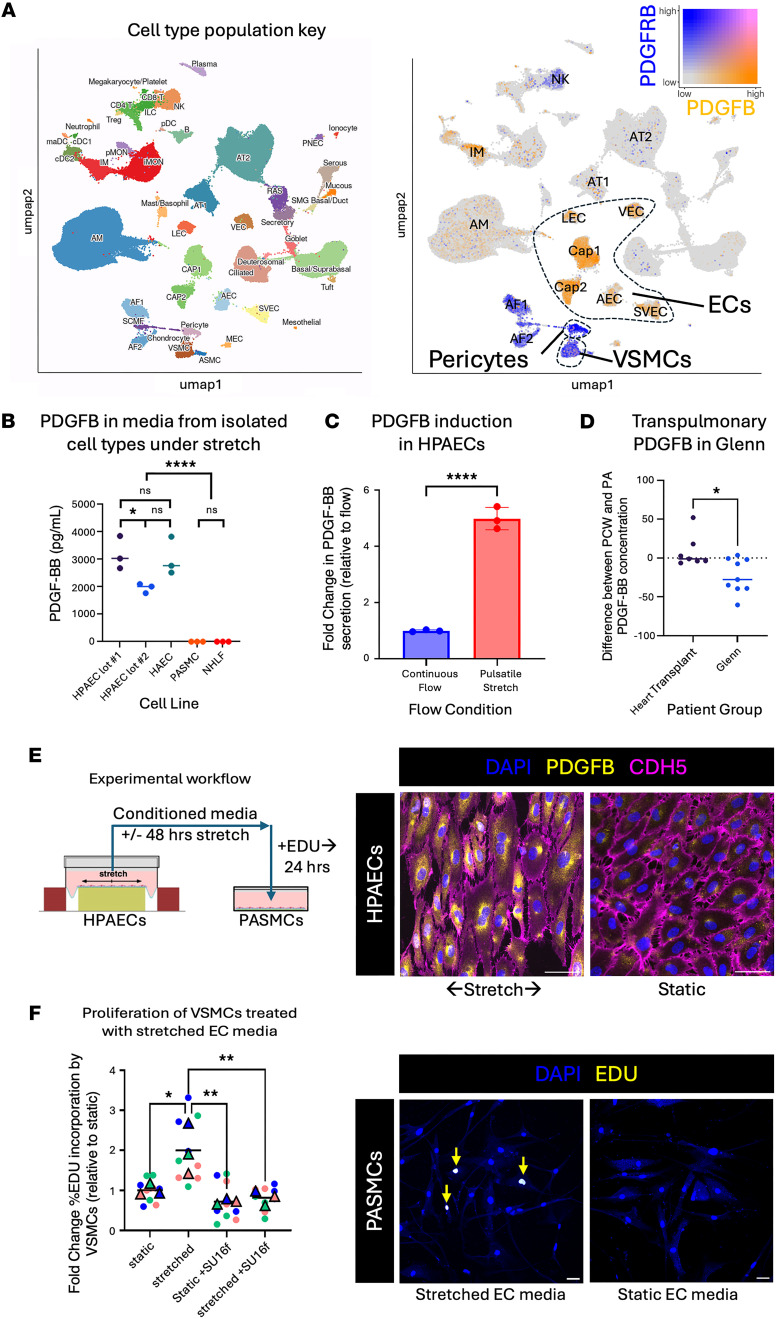
PDGFB expression in human lungs is EC specific, stretch induced, and stimulates vSMC proliferation. (**A**) Left: Cell-specific gene expression in human lung tissue was analyzed within the LungMAP single-cell RNA-seq data (347,970 cells). Right: Expression of PDGFB is high in ECs, with its receptor PDGFRβ found in pericytes and vSMCs. AEC, arterial endothelial cells; AF1, alveolar fibroblasts; AF2, adventitial fibroblast; AM, alveolar macrophage; IM, interstitial macrophage; LEC, lymphatic endothelial cells; NK, natural killer cells; SVEC, systemic vein endothelial cells; VEC, pulmonary venous endothelial cells. (**B**) Different human cell lines comprising the primary components of a blood vessel (endothelium, smooth muscle, fibroblasts) were exposed to pulsatile uniaxial 10% stretch at 1 Hz and only ECs secreted PDGFB into the media (as measured by ELISA). (**C**) Conditioned media from HPAECs placed under either 15 dyn/cm^2^ flow or 10% stretch at 1 Hz for 48 hours were assayed for PDGF-BB by ELISA, showing a 5-fold greater induction of PDGFB by stretch. (**D**) PDGFB measured by ELISA shows a significant reduction in plasma PDGFB from pulmonary artery to pulmonary vein (pulmonary capillary wedge sample, PCW) in plasma of Glenn patients compared with normal patients. (**E**) Left: Conditioned media from cultured HPAECs under stretch or static conditions were added to serum-starved (6 hours) PASMCs with or without SU-16f (a potent and selective small-molecule inhibitor of PDGFRβ), and EdU incorporation was measured after 24 hours. Right: Example immunofluorescent staining of PDGFB production by stretched ECs used for this experiment. (**F**) Left: EdU incorporation was greatest in PASMCs treated with stretched EC media, but the proliferative stimulus was blocked by SU-16f. Right: Example immunofluorescent staining of EdU incorporation by PASMCs. (**E** and **F**) Scale bar: 50 μm. *P ≤ 0.05; **P ≤ 0.01; ****P ≤ 0.0001 by unpaired, 2-tailed Student’s *t* test (**B** and **C**) or unpaired, 2-tailed Student’s *t* test with Welch’s correction (**D** and **F**).

As stretch accounted for the majority of pulsatility-regulated genes, we utilized published single-cell RNA-seq of human lung tissue (LungMAP; refs. 43, 44) to assess the cell-type-specific expression pattern of candidate genes (Supplemental Figure 6). We reasoned that narrowing the investigation to EC-specific genes shown to be in lung might identify endothelial factors that sense flow and normally respond by bolstering ECM or cellular components of the arterial media to increase resistance in proportion to hemodynamic load. *Mgp*, *Epcam*, *Cx3cl1*, and *Pdgfb* were highly expressed in a good proportion of ECs under stretch (Figure 3E), but we noted high EC specificity of the latter 2 genes. Analysis of the expression of these ligands and their receptors (*Cx3cl1R* and *Pdgfrb*, respectively) (Figure 4, A and B) underscored the pulsatility-induced crosstalk between ECs and perivascular cells (*Pdgfb*) (Figure 4A), and between ECs and immune cells (*Cx3cl1* and *Ccl14*) (Supplemental Figure 6). We focused on the PDGFB pathway, as PDGFB is (a) well known to promote vSMC proliferation and recruitment (30), (b) a key component of the GSEA for ECs under stretch (Figure 2), and (c) a significant hit in our differential gene expression (Figure 3), and further investigated its role in pulmonary vessels.

### Pulsatile stretch uniquely drives EC PDGFB secretion.

To determine whether stretch induction of PDGFB expression was specific to ECs or also present in other cellular components of the vessel wall (vSMCs or fibroblasts), we assessed the impact of stretch on different cell types found in the vessel wall. We applied cyclic 10% stretch to HPAECs from 2 different patients: human aortic ECs (HAECs), human pulmonary artery SMCs (PASMCs), and normal human lung fibroblasts (NHLFs). We found that pulsatile stretch robustly induced secretion of PDGFB exclusively from EC lines (Figure 4B). As PDGFB is known to be induced by flow (45), we compared PDGFB secretion from ECs under continuous flow (mimicking the venous condition) to pulsatile stretch (mimicking the arterial condition). Induction of PDGFB by pulsatile stretch was 5-fold higher than the induction by laminar, continuous flow (Figure 4C).

We next assessed whether we could detect EC secretion of PDGFB in human patients by comparing the concentration of PDGFB in pulmonary veins with the concentration in the PAs (transpulmonary gradient). We hypothesized that PDGFB would be accumulate in the blood throughout the pulsatile, arterial endothelium and thus be carried to the veins, where its concentration would be higher in normal patients. We anticipated that detection in circulating blood would be difficult, as PDGFB is secreted basally to the perivascular space, not apically into the blood stream (46). Testing the blood of patients with either normal (heart transplant patient with normal vascular anatomy) or Glenn anatomy revealed that normal patients maintain a stable concentration of PDGFB across the pulmonary vascular bed (Figure 4D). However, Glenn patients demonstrated a significant reduction in the transpulmonary gradient of PDGFB relative to normal patients (Figure 4D). These data support the idea that absence of pulsatility in Glenn patients impairs stretch-induced PDGFB secretion.

Lastly, we examined PDGFB expression in stretched versus static HPAECs (Figure 4E) by immunofluorescence, as well as functional impact of conditioned media from these cells on vSMCs. Stretched HPAECs displayed significant expression of PDGFB protein compared with static HPAECs (Figure 4E). When conditioned media from these stretched ECs were added to cultured PASMCs, it induced a significant, 2-fold increase in proliferation (Figure 4F). This effect was abrogated by the addition of the potent and highly specific small-molecule PDGFRβ inhibitor SU16f (Figure 4F). These data demonstrate that stretched ECs relay a functionally relevant PDGFB signal to vSMCs, stimulating their proliferation.

### Decreased PDGFB expression in pulmonary arteriole ECs of the Glenn rat model.

Given the finding that stretch induces PDGFB secretion from pulmonary ECs, we utilized our animal model of the Glenn surgery to test whether arteries receiving non-pulsatile flow exhibited decreased endothelial PDGFB synthesis in vivo. We performed an end-to-end anastomosis of the left SVC (L-SVC) and the left PA (LPA) (Supplemental Figure 7). This mimics the Glenn surgery, directing only non-pulsatile venous blood flow from the head and upper extremity into the PA. As previously described, these rats develop hypoxemia secondary to intrapulmonary arteriovenous shunting (38). The hypoxemia develops rapidly (after 2 weeks) and is progressive (to 6 months; ref. 38).

**Figure 5:**
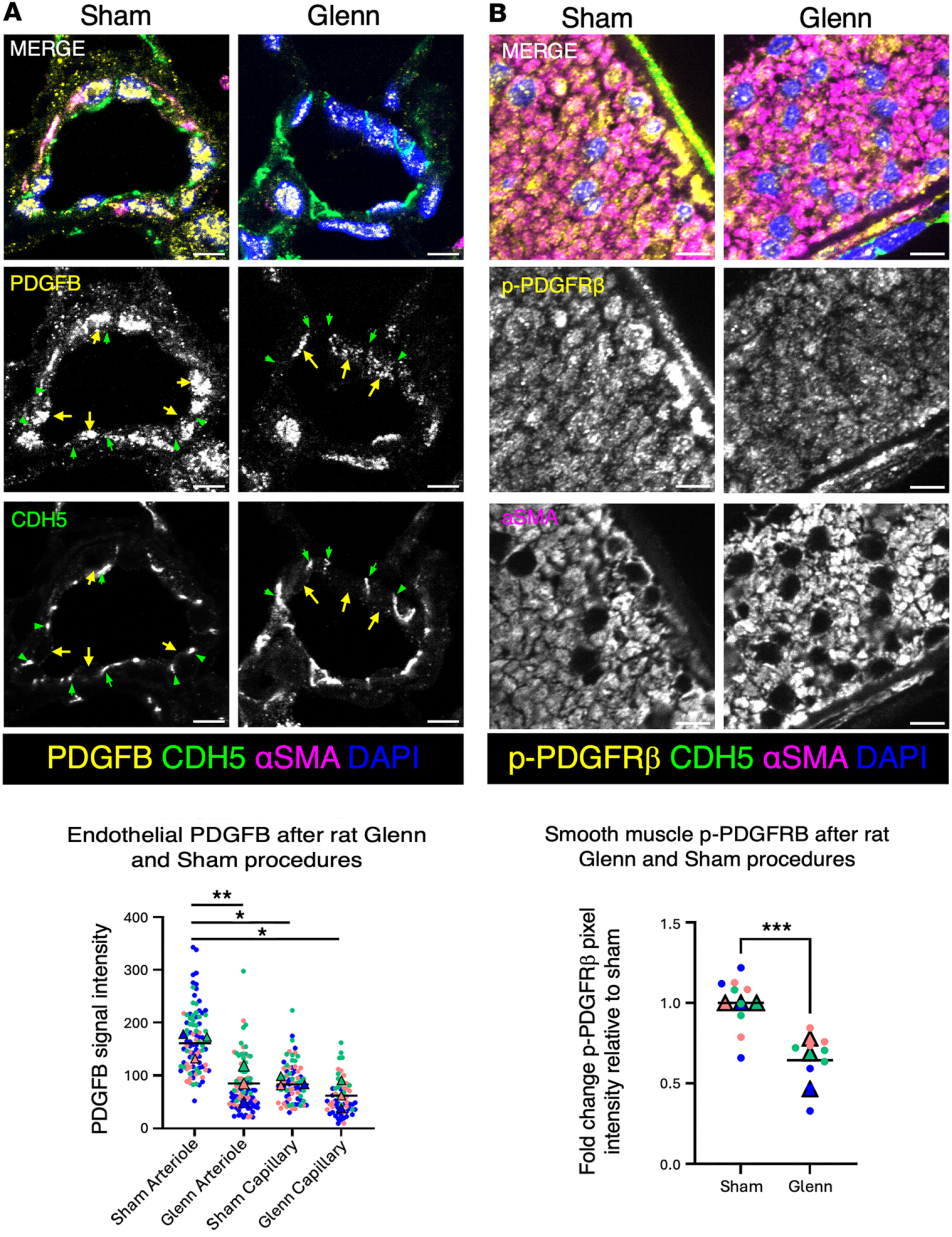
Loss of PDGFB in pulmonary endothelium and p-PDGFRβ in pulmonary vascular smooth muscle of rats after Glenn surgery. (**A**) Left: Representative immunofluorescent (IF) staining of pulmonary arterioles from left lung of sham- and Glenn-operated rats. Pulmonary arterioles were identified by presence of VECAD (cyan) and αSMA (pink), near lung periphery, and less than 50 μm in diameter. Scale bars: 5 μm. Right: Quantification of PDGFB IF staining in pulmonary arteriolar and capillary ECs in sham- (*n* = 91 arteriolar ECs, *n* = 74 capillary ECs) and Glenn-operated (*n* = 89 arteriolar ECs, *n* = 62 capillary ECs) rats demonstrates significant loss of EC-derived PDGFB in the Glenn lung. Five vessels each from the left lung of 3 sham and 3 Glenn rats were analyzed. (**B**) Left: Representative IF staining of proximal pulmonary arteries from left lung of sham- and Glenn-operated rats for p-PDGFRβ (yellow). Pulmonary arteries were identified as >100 μm in diameter, having ECs (CDH5, green), bounded by vascular smooth muscle (αSMA, pink), and by similarity in size and proximity to the largest airway in slices near the hilum of the lung. Scale bars: 5 μm. Right: Quantification of mean p-PDGFRβ staining intensity in αSMA^+^ cells (using the αSMA signal to mask the p-PDGFRβ channel) in the subendothelial medial layer in sham and Glenn rats (*n* = 3 vessels for each of *n* = 3 sham and *n* = 3 Glenn rats). *P ≤ 0.05; **P ≤ 0.01; ***P ≤ 0.001 by unpaired, 2-tailed Student’s *t* test with Welch’s correction (**A** and **B**).

To test whether rat arteries receiving non-pulsatile flow exhibited decreased PDGFB synthesis in their ECs, we focused on the distal lung arterioles — in the vicinity of where AVMs form in our patients, and where EC-specific expression of PDGFB is highest by single-cell RNA-seq (Figure 4A). To localize these arterioles and the PDGFB expression within the ECs, we used immunofluorescent staining with antibodies recognizing the endothelial junction marker VE-cadherin (CDH5), the vascular smooth muscle marker α-smooth muscle actin (αSMA), and the PDGFB protein (47). We found a significant reduction in endothelial PDGFB in arterioles of the Glenn rat (Figure 5A). Critically, we also noted loss of smooth muscle surrounding these distal arterial vessels in the Glenn. Quantification of the PDGFB immunofluorescence signal from 5 arterioles each from 3 sham- and 3 Glenn-operated left lungs revealed a significant decrease in endothelial PDGFB expression in the Glenn arterioles, but not in alveolar capillary ECs (Figure 5A).

We next asked whether phosphorylation (activation) of PDGFRβ was also reduced in vivo in the Glenn vSMCs, downstream of the reduction of EC-derived PDGFB in the Glenn. We were unable to accurately test this in distal vessels due to patchy vSMC coverage of the arterioles in the distal lung, and so we examined the proximal PAs. We compared the mean p-PDGFRβ signal in the media of the vessel wall (media, masking with the αSMA signal). Analyzing 3 fields of view of the most proximal branch PAs from each of 3 sham and 3 Glenn rats, we noted a significant reduction in p-PDGFRβ in the vSMCs of Glenn PAs (Figure 5B).

### Glenn rat displays reduced PA smooth muscle.

**Figure 6:**
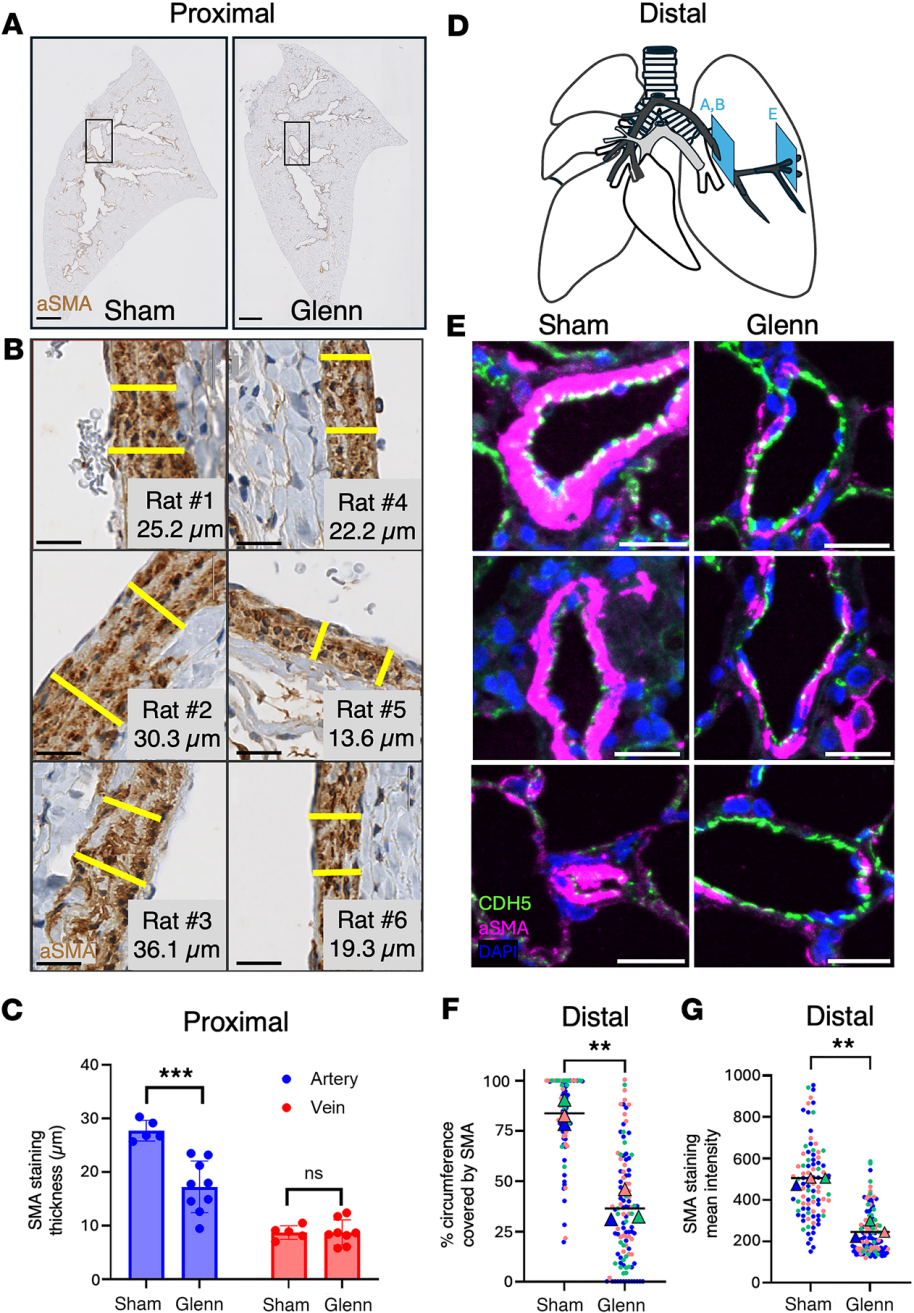
Loss of smooth muscle layer in pulmonary arteries of rats after Glenn surgery. (**A**) Example sagittal sections of lung tissue from sham- and Glenn-operated rats, stained by immunohistochemistry for αSMA. Black boxes show typical perihilar location of analysis of the most proximal portions of pulmonary arteries examined. Scale bars: 2 mm. (**B**) Left: Example images of αSMA^+^ media thickness from 3 different sham-operated rats. Ten distributed measurements per vessel were taken and the mean thickness was used; yellow bars show example measurements. Right: Example images of αSMA^+^ media thickness from 3 different Glenn rats. Scale bars: 20 μm. (**C**) Quantification of αSMA^+^ tunica media from proximal arteries versus veins. (**D**) Schematic showing approximate location of pulmonary vessels assayed in **A**, **B**, and **E**. (**E**) Examples of images of αSMA^+^ media thickness in distal arterioles from 3 different sham (left panels) and Glenn (right panels). Scale bar: 25 μm. Artery regions analyzed were chosen as those vessels near lung periphery and <50 μm in size. Distal vessels <1 mm were identified using immunofluorescent staining for CDH5 (green) in sham (*n* = 3 rats, *n* = 42 vessels) and Glenn (*n* = 3 rats, *n* = 56 vessels) rat lungs. (**F** and **G**) Staining of αSMA (pink) was used to quantify the circumferential coverage (**F**) and intensity (**G**) αSMA surrounding these arterioles. **P ≤ 0.01; ***P ≤ 0.001 by unpaired, 2-tailed Student’s *t* test with Welch’s correction (**C**, **F**, and **G**).

We hypothesized that a further consequence of reduced PDGFB in pulmonary ECs under the non-pulsatile Glenn hemodynamics would be loss of the smooth muscle layer. We analyzed sagittal sections of the left lung of 9 Glenn rats and 5 sham-operated rats and performed immunohistochemistry for αSMA. Strikingly, we found a significant decrease in arterial smooth muscle thickness (αSMA^+^ cells) (Figure 6, A and B), while venous smooth muscle thickness remained unchanged (Figure 6C). We observed this reduction in vascular wall thickness in both the largest PAs (Figure 6, A–C), as well as the distal arterioles (measured by immunofluorescence intensity), less than 0.1 mm in diameter (Figure 6, D–G).

## Discussion

**Figure 7:**
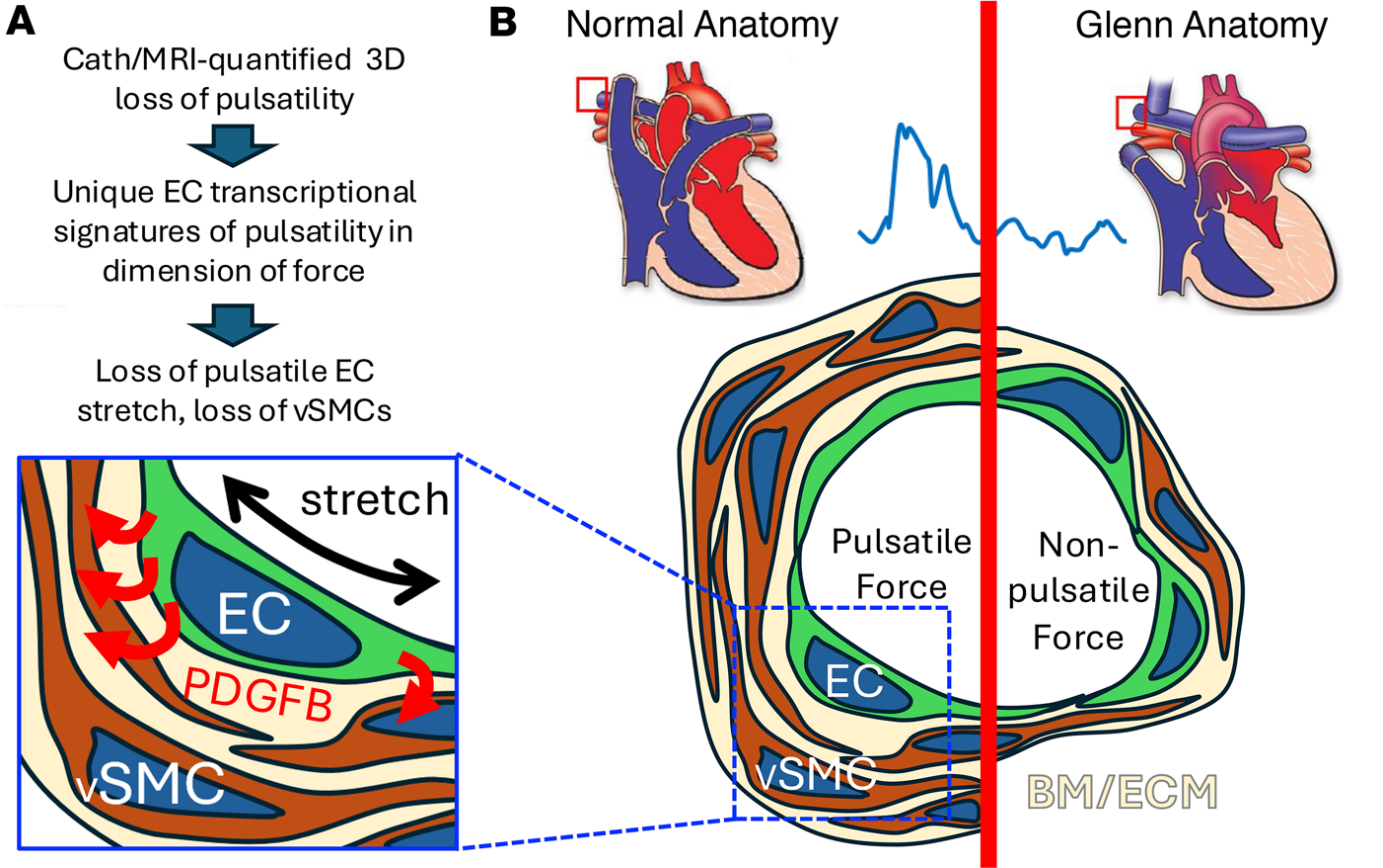
Model for stretch-induced EC secretion of PDGFB stimulating smooth muscle vessel support. (**A**) Overview of experimental results. (**B**) Comparison of findings between pulsatile and non-pulsatile conditions. Example pressure waveform from cardiac catheter shown by blue line. Red boxes indicate region of pulmonary arteries analyzed in patients with either normal lung vascular architecture or Glenn anatomy. Blue box area shows increased PDGFB secretion from ECs (green) following blood circulation–induced stretch. vSMC, vascular smooth muscle cell (brown); BM, basement membrane; ECM, extracellular matrix (beige).

In this study, we identify hemodynamic pulsatility as critical to maintenance of the PA architecture (Figure 7). We show that, in patients with Glenn circulation, shunted pulmonary blood flow from the SVC directly to the PA exerts no pulsatile stretch on the PA. We model altered hemodynamics in cultured PA ECs exposed to each dimension of force and use transcriptomic analysis to demonstrate that each elicits distinct molecular signatures. We find that pulsatile stretch induces significant EC secretion of PDGFB, known to mediate recruitment of vSMCs to blood vessel endothelium (30, 31). As lung biopsies from living patients were not obtainable, we used our rat model of the Glenn circulation to investigate the effect of pulsatility loss in vivo. We show that non-pulsatile, venous-like flow within an arterial vessel results in loss of PDGFB protein from ECs, decreased p-PDGFRβ in VSMCs, and concomitant thinning of the vascular mural wall. Our complementary in vitro and in vivo findings identify a target signaling pathway for novel therapeutic approaches to treat the diverse VMs that occur in Glenn patients.

Despite clinical appreciation of the fact that Glenn patients experience venous-type, non-pulsatile blood flow in PAs, and even though pulsatility loss was the first hypothesized mechanism for Glenn PAVMs (48, 49), pulsatility loss has never been formally documented in 3 dimensions to the best of our knowledge. Until now, studies comparing surgical outcomes of Glenn patients have only occasionally been able to report some dimensions of pulsatility loss (9, 50), although most studies report none (51–57). This clinical knowledge gap has limited our understanding of the impact of pulsatility within these patients. Our use of cath/MRI ensures that these interdependent forces are measured simultaneously, providing with high temporal accuracy the first complete 3D view to our knowledge of pulsatility loss in the Glenn PAs.

Our pulsatility metrics merit 2 points of discussion. First, we utilize flow velocities as a surrogate for wall shear stress (WSS), the direct hemodynamic force applied to the endothelium. As WSS depends directly on the velocity gradient, velocity is a reasonable approximation for this investigation. Given the limited availability and accuracy of WSS measurements with cardiac MRI, use of velocity instead of the highly regionally variable WSS supports an easier and more rapid clinical application. Second, the relationship between pressure and stretch (vascular compliance) was not directly tested in our study. The issue of compliance will be a critical area of investigation in the future, as loss of normal endothelial function and medial structure in many other clinical conditions (pulmonary hypertension, congenital aortopathies) results in abnormal gain (congenital aortopathies) or loss (pulmonary hypertension, aortic calcification, carotid atherosclerosis) of compliance (34). Vascular compliance also directly relates to the progression of pulsatility into the furthest branches of the arterial tree.

However, estimating the magnitude of pulsatile forces across the pulmonary vascular tree is difficult. Arterial pulsatility in normal lung vessels is likely significantly diminished as it reaches distal capillaries. Dynamic measurement of distal pulsatility of stretch in sub-millimeter vessels in patients is technically challenging and could not be directly assessed by our methods. In a normal compliant vessel, proximal stretch acts as a capacitor, absorbing and lessening distal pulsatility, a phenomenon termed the “windkessel effect” (58). This model suggests a reduction in pulsatility in the normal distal microvasculature.

Our proposed mechanism — that pulsatile stretch of ECs stimulates PDGFB followed by smooth muscle coverage — is supported by the observation that normal arterial smooth muscle thins as vessels branch further into the lung (12). Critically, EC-specific ablation of PDGFB leads to loss of mural cell coverage in retinal vessels and promotes shunt development in murine lungs (34). PDGFB was originally described as flow responsive (45), and later as stretch responsive (59), although our in vitro work shows that stretch elicited 5-fold more PDGFB secretion than flow. The opportunity now arises to characterize the relative contribution of flow (LSS) and stretch to PDGFB secretion in these distal vessels. We also recognize that the presence of respiration-driven alveolar stretch (at a much lower frequency) may also contribute to distal pulsatility (60, 61). In all, these studies underscore the importance of pulsatility throughout the entire arterial network and bring up new questions about the impact of biomechanical cues on the lung vasculature.

How do ECs sense hemodynamic forces in lung vessels? We previously hypothesized that endothelial cilia could be involved in sensing pulsatility of flow (62) but dismissed this possibility in the mammalian lung, when we showed that pulmonary ECs are largely devoid of primary cilia (63). Endothelial mechanosensitive components have been well described and are present on lung ECs. Notch, CDH5, and many other cell-surface proteins, ECM, and cytoskeletal elements are all required for proper sensing of flow or stretch (22). In particular, the protein complex of PECAM1, CDH5, VEGFR2/3, and PLXND1 plays a critical role in sensing force (21), while proteins like LPHN2 and others coordinate downstream responses (64). In our data, we see significant upregulation of *Plxnd1*, as well as nearly 2-fold increases in *Cdh5*, *Kdr*, and *Lphn2* (data not shown) under pulsatile pressure conditions, but not pulsatile stretch or LSS, suggesting a possible role for pulsatile pressure in priming the endothelium to respond to force. Our study thus highlights the need to consider each dimension of force separately, as we continue to evaluate the role that pulsatility plays in the maintenance of the normal vascular architecture.

Other cells of the vascular wall, such as vSMCs, also certainly sense biomechanical forces. vSMCs experience stretch similarly to ECs but not LSS, as they are not in direct contact with blood flow. Interestingly, vSMCs respond differently to stretch than ECs, aligning parallel to stretch in vivo, rather than perpendicular like ECs (65). In our study and in publicly available single-cell RNA-seq from human lung, we also find that vSMCs do not secrete PDGFB. VSMCs in Glenn pulmonary vessels do, however, show reduced PDGFRβ phosphorylation, demonstrating reduction in EC-vSMC crosstalk. We also note that vSMCs from Glenn rats do not show changes in standard vSMC genes such as *Myh11*, *Tagln*, *Myocd*, *Cnn1*, and *Pdgfrb* (data not shown). This does not rule out, however, the possible contribution of vSMCs to the ECM, especially regarding the changes in pulmonary vascular resistance and vessel stiffening that so critically drive patient outcomes. Future studies will be necessary to investigate the responses of vascular smooth muscle to pulsatile stretch.

While we focused here on *Pdgfb*, we also note that under pulsatile flow there was a significant upregulation of *Col1a2*, a major component of the vascular wall. Loss of *Col1a2* is associated with vasculopathy and aortic dilation (66), suggesting that pulsatility of stretch likely drives other EC contributions to the structure of the arterial wall beyond the recruitment of vSMCs. Regulation of the ECM is a logical complementary function of pulsatility, as the scaffold for the vSMCs and the internal elastic lamina of the largest arteries are unique features of overall arterial structure.

In addition, we also noted the upregulation of CX3CL1 in pulsatile-stretched HPAECs. CX3CL1 is a well-known chemokine that increases leukocyte recruitment and adhesion to ECs (67, 68). Multiple other chemokines (including CCL2 and CCL14) are regulated by pulsatility in our in vitro system. The role of the immune system in the vascular complications of Glenn circulation is a wholly unappreciated factor; however, previous work in hereditary AVMs identified accumulation of leukocytes in brain AVMs and skin telangiectasias (69–71). Collectively, these findings suggest that pulsatile stretch may impact EC-leukocyte crosstalk in pathogenesis of VMs.

As we seek to translate our findings to our patients, we noted a significant reduction in the transpulmonary gradient of PDGFB in Glenn patients relative to patients with normal anatomy. This finding could be due to several reasons: (a) steady rate of uptake/decay of plasma PDGFB coupled with decreased production in the Glenn PAs, (b) arteriovenous shunting that bypasses and thus does not stimulate PDGFB production from the capillary bed, or (c) another unforeseen reason. It remains to be seen whether PDGFB modulation can impact the development of AVMs or the formation of collateral vessels in patients with a Glenn or Fontan circulation.

In summary, we show that the pulsatility of hemodynamic forces in blood vessels is sensed by the endothelium uniquely in each dimension of force. Our data support a model whereby pulsatile stretch induces endothelium to secrete PDGFB, signaling for support from vascular smooth muscle. Indeed, the architecture and cellular composition of the vessel wall change significantly upon loss of pulsatility in the Glenn, with thinning of the vascular wall, thereby shifting the arterial vascular wall structure toward that of a vein, likely impacting downstream endothelium. Together, this work underscores the critical importance of proper blood flow dynamics for the stability of vascular architecture and function. In addition, it points to biomechanical signaling pathways as potential novel targets for therapeutic intervention in patients with pulsatility loss, such as in the Glenn circulation. We seek to better understand and address the mechanistic underpinnings of vascular defects that occur during single-ventricle palliation and anticipate that our data on pulsatility will provide the foundation for improving clinical outcomes for children that undergo these necessary surgeries.

## Methods

### Sex as a biological variable.

Both male and female patients were included in the study (see Supplemental Table 1) Findings are expected to be relevant for both male and female patients, as the vascular connections and thus the hemodynamic state in the Glenn is similar in both male and female patients.

### Cath/MRI.

Cath/MRI was performed on a Philips XMR setup (Phillips Ingenia Evolution) (72). This consists of a 1.5 T MRI scanner and a BV Pulsera or Allura Clarity or Ingenia (Philips) cardiac x-ray unit. An appropriately sized balloon wedge catheter (Arrow Intl.) and receiver coil were used depending on the weight of the patient.

All studies were conducted under general anesthesia based on clinical need. All Glenn patients with a single functional ventricle were considered for inclusion in the study. Patients were excluded if they had pulmonary blood flow in addition to the flow provided by the Glenn anastomosis.

Pulsatility was assessed by pulse difference, which was defined as the difference between maximum and minimum value for each parameter of force. Pulsatility index was not selected due to the potential for obscuring differences between patients (see Supplemental Table 2). For each 2D cross section of the RPA obtained, we obtained a mean velocity (averaged over the cross section of the vessel). These data were obtained over 1 to 2 minutes of “free breathing” using phase-contrast velocity-encoded cine MRI. Sequence parameters included 40 cardiac phases, TE/TR = 2.7/4.4 ms (TE = echo time, TR = repetition time), with 2 signal averages, 1.5–2 mm × 1.5–2 mm × 6–8 mm resolution, SENSE acceleration factor = 2, and with the velocity encoding gradient set to 25% above the expected maximum velocity in each vessel. Vendor-provided background phase correction was used. Artificial intelligence–assisted segmentation using Circle (Circle Cardiovascular Imaging, CVI42, v6.1.2) was performed to obtain the maximum and minimum area, from which an idealized circumference was calculated. Pressure tracings were obtained on a standard Siemens Sensis Hemodynamic recording system (Siemens Healthineers), using a 5- or 6-French Arrow, Balloon Wedge-Pressure Catheter (Teleflex Medical Headquarter International) located in the proximal RPA.

### In vitro cell-based assays of pulsatile force.

Pulsatile force was applied to confluent monolayers of HPAECs (Lonza) for 48 hours under each condition. Pulsatile (1 Hz, 15 dyn/cm^2^) and continuous (15 dyn/cm^2^) shear was applied using the Ibidi system (Ibidi). Pulsatile (25–5 mmHg) and continuous (25 mmHg) pressure was applied using a custom modification to the Ibidi system whereby cells were alternately (1 Hz) exposed to columns of culture media to deliver the desired hydrostatic pressure. Pressure readings were tested and confirmed the using the Siemens Sensis Hemodynamic recording system listed above. Pulsatile stretch was applied using Uniflex plates (1 Hz, 1 hour of 3% stretch for acclimation followed by 47 hours of 10% stretch) as a part of the Flexcell FX-6000 system (Flexcell International). Ibidi channels were precoated with the proprietary Ibi-treat to promote cell adhesion, and Flexcell Uniflex plates were coated with 0.1% gelatin to promote cell adhesion.

All experiments utilized at least 3 biological replicates, using separate aliquots of HPAECs from 2 different donors. RNA was extracted using an RNeasy Plus kit (Qiagen) prior to bulk RNA-seq. Library generation and bulk RNA-seq were carried out by the McDermott Next Generation Sequencing core facility at UT Southwestern Medical Center, using an Illumina NextSeq 2000. Processed sequencing data (using iGenomes annotations) were provided by the core, and a TPM cutoff of 10 was applied (if any test condition resulted in a gene’s TPM >10, the gene was included in our analysis). Volcano plots were generated using VolcaNoseR (73). PCA plots of each condition are shown in Supplemental Figure 4.

For pathway analysis, GSEA was performed for sets curated in MsigDb (PMID 26771021) using the Bioconductor fgsea package (http://bioconductor.org/packages/fgsea/). To simultaneously account for both the magnitude and significance of the measured effects, genes were ranked by the quotient of log(fold change) and adjusted *P* value.

### Human lung single-cell RNA-seq.

The LungMAP Human Lung CellRef atlas was utilized via their online portal (https://www.lungmap.net/ Accessed March 15, 2025.) (43, 44). This dataset includes 148 normal human lung samples from 104 donors, including child, adolescent, and adult tissue. Tissue types include parenchyma, trachea, bronchi, bronchus, and small airways. We included 347,970 cells from the dataset in our analysis. Both ShinyCell (https://app.lungmap.net/app/shinycell-human-lung-cellref) and CELL×GENE (https://cellxgene.cziscience.com/e/eb499fd8-7000-419f-8854-926b9b61b11f.cxg/) were utilized for visualization of cell-specific gene expression (dataset ID: LMEX0000004396).

### Measurement of PDGFB secretion in vitro.

Cultured HPAECs, HAECs (Lonza), human PASMCs (Lonza), and NHLFs (Lonza) were plated onto Flexcell Uniflex plates (precoated with collagen I). Each cell line was grown in the recommended cell culture media, obtained from Lonza; HPAECs and HAECs were grown in EGM-2, PASMCs in SmGM-2, and NHLFs in FGM-2. Cells were grown to confluence and then subjected to pulsatile stretch for 48 hours, as above. Media were collected and PDGF-BB was measured by ELISA (R&D Systems, DY220). For comparison to ECs under flow, media were obtained from confluent HPAECs in 6-well plates under continuous flow at 15 dyn/cm^2^ shear stress on a rotational flow apparatus (Thermo Fisher Scientific, model no. 88881101).

For proliferation experiments with conditioned media from HPAECs, culture media were serum free to avoid the confounding proliferative effects of fetal bovine serum. Cell death was monitored by visual inspection (cells maintained at >80% confluence) during the experiment, and conditioned media were centrifuged at 10,000*g* for 15 minutes prior to aliquoting for the assay. SMCs were starved for 6 hours prior to treatment with conditioned EC media to synchronize the cell cycle. EdU was added to the conditioned media at the time of media addition to the vSMCs. EdU incorporation was identified using the Click-iT EdU Cell Proliferation Kit (Invitrogen, C100338). SU-16f (MilliporeSigma, AABH97CD6FE8) was applied where indicated to a final in-well concentration of 1 μM, targeting 100 times the reported IC_50_ for PDGFRβ but keeping it below the threshold for significant off-target effects on VEGFR2 signaling. Each condition of the assay was performed with 3 biological replicates (individually stretched samples of ECs) and 3 technical replicates (individual wells of vSMCs treated with EC media).

For measurement of the transpulmonary gradient of PDGF-BB (here termed PDGFB) in human plasma, samples were obtained directly from Glenn and heart transplant patients in the course of regularly indicated cardiac catheterization. Samples were obtained from the precapillary blood (RPA) and the postcapillary blood (right pulmonary wedge position, with the catheter in the distal PA with balloon inflated and the sample drawn through the end hole) in each patient. The nature of each sample as pre- or postcapillary was then confirmed by prompt measurement of oxygen saturation, and only fully oxygenated samples were used for the analysis. All samples were obtained in K2-EDTA purple-top tubes, placed on ice, and promptly spun down for plasma isolation. Samples were stored at –80°C up to 2 years maximum prior to measurement by ELISA as above.

### Surgical rat model of Glenn circulation.

The rat surgical model of Glenn circulation was previously described in detail (38). Briefly, we performed an end-to-end cavopulmonary anastomosis between the L-SVC and the LPA. This was previously shown to result in progressive impairment (beginning by 2 weeks) in oxygenation in tandem with the development of intrapulmonary arteriovenous shunts, as detected by both bubble echocardiography and microsphere injection.

Adult Sprague-Dawley rats (male and female, 6–12 weeks of age) were used for all experiments (Taconic). All rats were housed in the Biomedical Research Center at the Medical College of Wisconsin with access to standard chow diet (PicoLab Lab Diet, 5L0D), water ad libitum, and maintained in a 12-hour light/dark cycle. All surgical procedures were performed under isoflurane anesthesia (1%–3%).

### Tissue preparation and immunohistochemistry/immunofluorescence.

Lungs were harvested at 2 and 6 months after surgery for these experiments. Lungs were inflated to 10 mmHg for 10 minutes with 10% neutral buffered formalin to promote normal alveolar architecture in sections, followed by overnight fixation in 10% neutral buffered formalin. Formalin-fixed, paraffin-embedded sections were prepared using the left lung of the Glenn- and sham-operated animals. Immunohistochemical staining was done for αSMA (Abcam, ab7817) using the Leica Bond Rx automated staining platform with Leica BOND Polymer Refine Detection kit (Leica, DS9800) per manufacturer instructions, using standard labeled streptavidin-biotin detection with secondary antibody (Jackson Immuno Research Labs, biotinylated donkey anti-mouse, 715-066-151; 1:500), streptavidin HRP (Vector Laboratories, SA-5004), and DAB chromogen (BioCare, BDB2004).

Slides were scanned using a Hamamatsu HT whole slide scanner and analyzed with QuPath (Edinburgh, v0.5.1). The file names were randomized and blinded prior to analysis. Due to the possibility of changes in vessel size between sham- and Glenn-operated rates, measurements were taken from the most proximal pulmonary arteries and veins to ensure a valid comparison between samples. Arteries were differentiated from veins in consultation with a trained pathologist, by their classic histological characteristics and anatomical proximity to the mainstem bronchi. Slides were not utilized for measurement if no major bronchi or large vessels could be identified. Measurements were taken only in areas where the thickness was uniform and the ECs formed a clear monolayer, to avoid artifactual error from the angle of the slice. Ten measurements of each large vessel (artery or vein, Glenn or sham operated) were obtained, and the mean measurement value was used for analysis.

We used immunofluorescent staining and confocal microscopy to identify distal vessels for quantification of endothelial PDGFB protein and perivascular smooth muscle. Lung sections were prepared as above. Sections were permeabilized for 10 minutes in 0.2% Triton X-100 in PBS, then subjected to antigen retrieval with R-buffer B in a 2100 Retriever (Electron Microscopy Sciences). Slides were blocked with CAS-block (Invitrogen), and primary antibody incubations were carried out at 4°C overnight. Primary antibodies were as follows: VE-cadherin/CDH5 (R&D Systems, AF1002; 1:100), PDGFB (Santa Cruz Biotechnology, sc-365805; 1:100), and αSMA-APC (Abcam, ab225143; 1:400). Imaging was performed on a Nikon CSU-W1 dual-camera inverted spinning-disc confocal microscope. Vessels were chosen as <50 μm and >20 μm in diameter, with a clear lumen and apical VE-cadherin staining visible. Images were analyzed in Fiji (https://imagej.net/software/fiji/downloads) as follows: the percentage of the circumference of each vessel covered by αSMA staining was calculated, and a mask was made of the pixels positive for αSMA staining in the sub-endothelial region and the mean pixel intensity of those pixels was calculated.

### Statistics.

Sample sizes were based on patient availability and standards in the field. Data were both analyzed and plotted in GraphPad Prism 10.2.3. Descriptive statistics (mean, standard deviation, median, min, and max) were employed for summarizing demographic, hemodynamic, and clinical variables. Statistical tests (indicated in the relevant methods sections and figure legends) utilized were the parametric unpaired Student’s *t* test and the 1-way ANOVA with Tukey’s multiple-comparison test. Statistical significance was defined as *P* value of less than 0.05. Superplots were made with individual colors representing biological replicates (individual rats), and the mean of the averages of each biological replicate is shown (74). Cell images were analyzed in Fiji. Figures and models were made using Microsoft PowerPoint, data were partially analyzed in Microsoft Excel, and text was written in Microsoft Word.

### Study approval.

After recruitment and obtaining written informed consent for the MRI studies, data were recorded in sequence of the patient’s referral for the procedure under a University of Texas (UT) Southwestern IRB–approved study (STU 032016-009) in 2023–2024. To obtain human plasma, patients provided written informed consent for the UT Southwestern IRB–approved study (UTSW-2020-0047). All experimental protocols involving animals were approved by the Medical College of Wisconsin Institutional Animal Care and Use Committee prior to initiation of experimental protocols (Animal Use Agreement no. 7731).

### Data availability.

All sequencing data that support the findings of this study have been deposited in the NCBI Gene Expression Omnibus (GEO) and are accessible through the GEO accession number GSE298790. All other relevant data are available from the corresponding author on request.

## Author contributions

SS, TH, MF, and OC conceptualized the study. SS, TH, MF, SVR, ADS, and OC developed the methodology. SS, LT, TW, SVR, CPC, and OC conducted experiments. SS, TW, ADS, TC, and OC provided resources. SS, MAC, LT, CPC, MLIA, and TH curated data. SS and OC wrote the original draft of the manuscript, which was reviewed and edited by SS, TH, ADS, MLIA, and OC. MAC and SS generated figures. SS and OC supervised the study and provided project administration. SS, TC, ADS, and OC acquired funding.

## Conflict of interest

The authors have declared that no conflict of interest exists.

## Funding support

- American Thoracic Society/Alveolar Capillary Dysplasia (ACDMPV) Research Grant 23-24PACDA12 (to SS).
- National Heart Lung and Blood Institute grants 1R01HL-175575 (to MLIA), K08HL157510 (to ADS), and HL113498 and HL126518 (to OC).
- National Academy of Science, Engineering, and Medicine Ford Foundation Dissertation Fellowship (to MAC).
- NIH grants R01DK127634 and RC2 DK125960 (to TC).
- Cancer Prevention Research Institute of Texas grant RP220201 (to TC).
- Foundation Leducq grant 21CVD03 (to MLIA and OC).
- National Heart, Lung, and Blood Institute grants U01-HL144861 (to the LungMAP Human Tissue Core) and U24-HL148865 (to the LungMAP Data Coordinating Center).

## Supplementary Material

Supplemental data

Supporting data values

## Version 1 05/08/2026

Electronic publication
