# Supplementary material for: Pulsatile flow dynamics maintain pulmonary artery architecture
Source: JCI Insight. 2026 May 8;11(9):e201797. doi: 10.1172/jci.insight.201797 (PMC13232008; doi:10.1172/jci.insight.201797)
Supplement: Supplemental data [file jciinsight-11-201797-s140.pdf]

Supplemental Figures and Tables

Supplemental Fig 1: Comparison of arterial and venous characteristics highlights the definitional importance of pulsatility

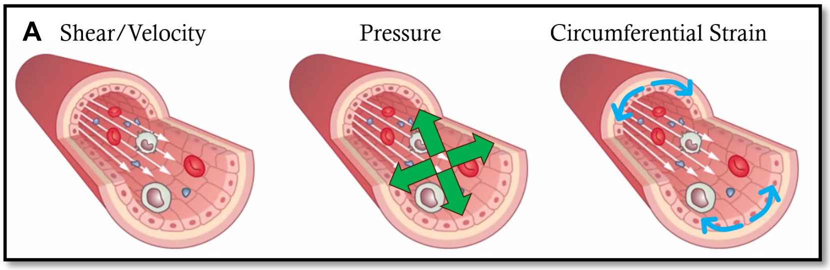

B		Systemic		Pulmonary		Umbilical	
		Artery	Vein	Artery	Vein	Artery	Vein
Hemodynamic Forces	Pulsatility	YES	NO	YES	NO	YES	NO
	Mean Pressure (mm Hg)	70	4	12	6	50	20
	Peak Velocity (cm/s)	100	15	75	40	45	15
Gas Transport	PO2	100	40	40	100	20	30
	PCO2	40	45	45	40	50	40
	pH	7.4	7.36	7.36	7.4	7.27	7.35
	Hgb Saturation	100%	70%	70%	100%	50%	70%
	Hgb Isoforms	HgbA/B	HgbA/B	HgbA/B	HgbA/B	HgbF	HgbF
Histological Features	Intima	+	+	+	+	+	+
	Internal Elastic Lamina	++	+	++	+	-	-
	Media (VSMCs)	+++	+	+++	+	+++	+
	Adventitia	++	+	++	+	++	+
	Forms Valves	NO	YES	NO	NO	NO	NO

Supplemental Fig 1: Comparison of arterial and venous characteristics highlights the definitional importance of pulsatility. (A) Three dimensions of hemodynamic force on endothelial cells. **(B)** Comparison of key features of the three major anatomical sets of arteries and veins. All values are approximate. Pulsatility is common to all arteries despite their pressure, oxygenation, or peak shear stress/velocity.

Supplemental Figure 2: Comparison of baseline transcriptome of key endothelial genes under static conditions

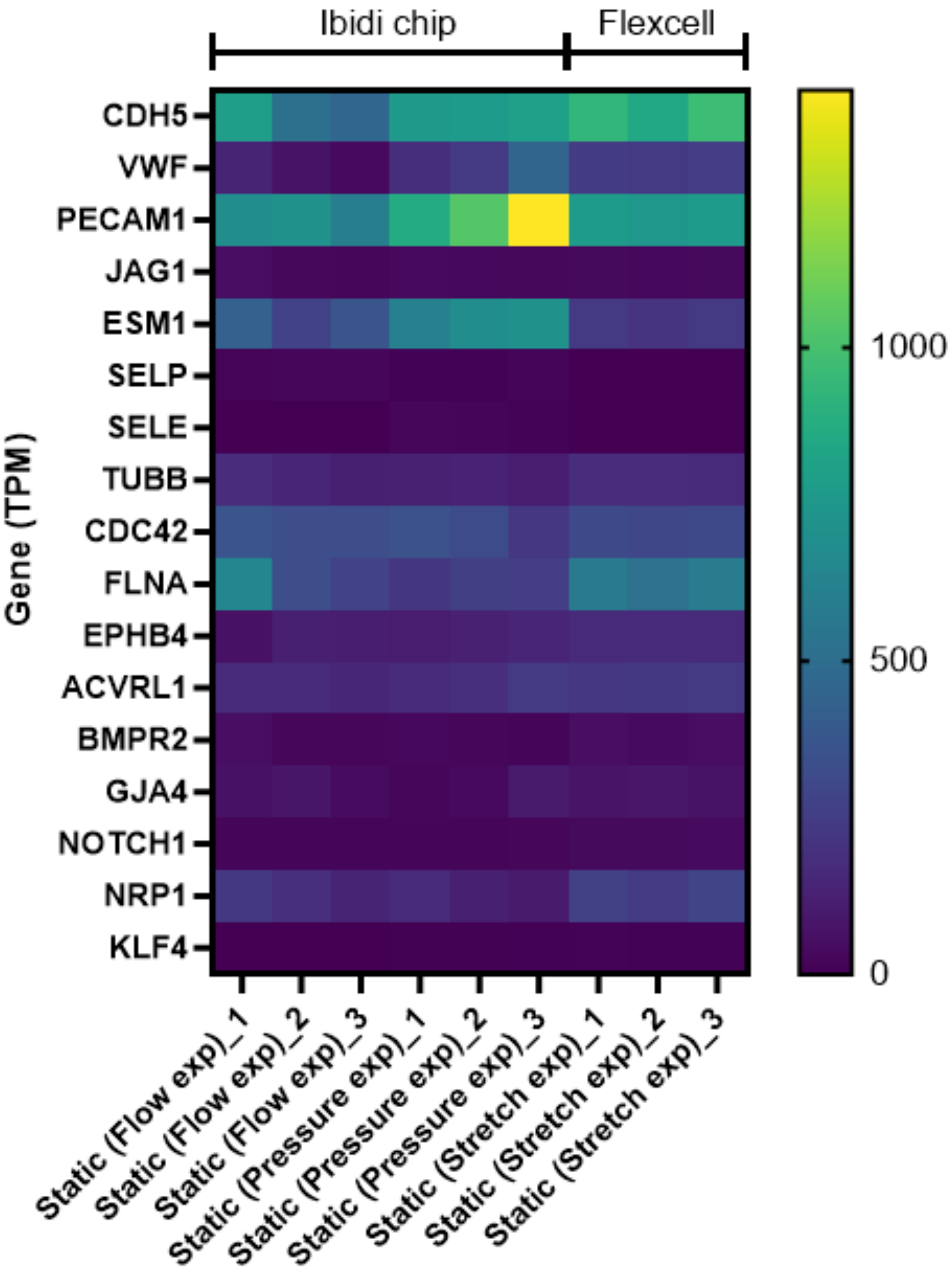

Supplemental Figure 2: Comparison of baseline transcriptome of key endothelial genes under static conditions. Heatmap comparing baseline expression of key endothelial cell genes shows that despite being seeded on different substrates, endothelial cells used in our experiments did not vary in their baseline conditions prior to the application of pulsatile or non-pulsatile force.

Supplemental Fig 3: Lung cell-specific expression of stretch- induced genes

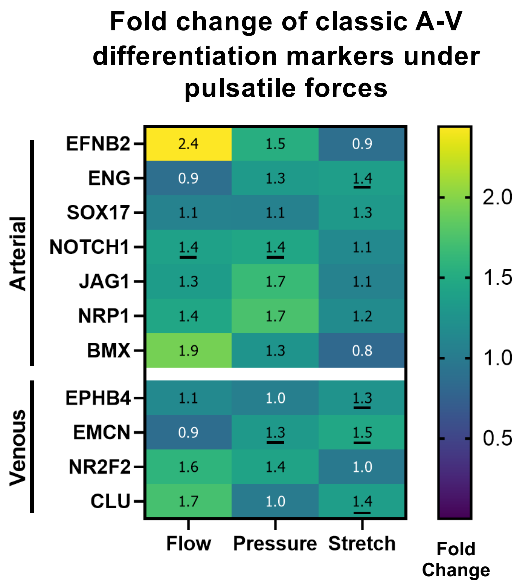

Supplemental Fig 3: Lung cell-specific expression of stretch- induced genes. Fold change (pulsatile/non-pulsatile) of any classic EC arteriovenous differentiation markers by bulk RNAseq. Color scale denotes fold change, exact number in each box. Significant changes (p-values < 0.05) are underlined.

Supplemental Figure 4: Impact of pulsatility on significantly regulated genes

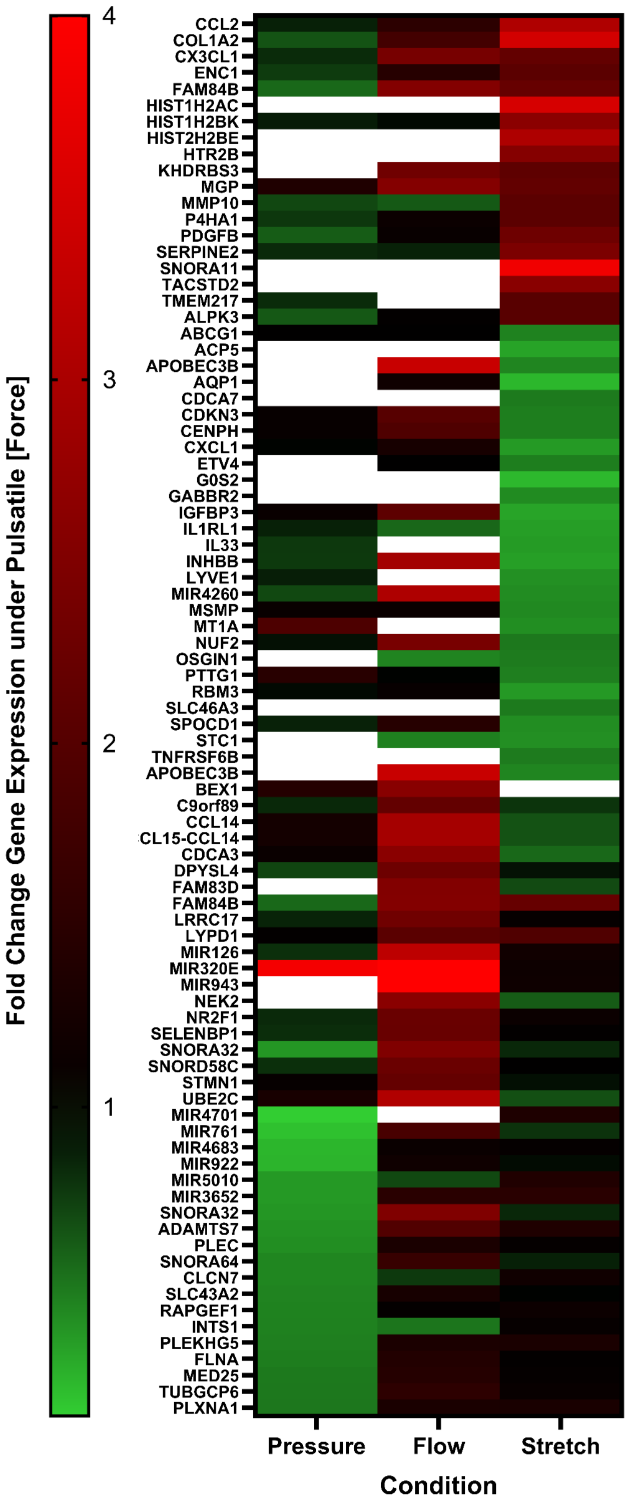

Supplemental Figure 4: Impact of pulsatility on significantly upregulated genes. (Alternate visualization of data in figure 3) Heatmap showing fold change in gene expression under different dimensions of force, comparing pulsatile to non-pulsatile conditions. White boxes indicate gene was excluded from analysis for that condition due to low expression (TPM < 10 for that gene under both pulsatile and non-pulsatile conditions). Red/Green bar at right indicates fold change

Supplemental Fig 5: PCA plots of bulk RNAseq data

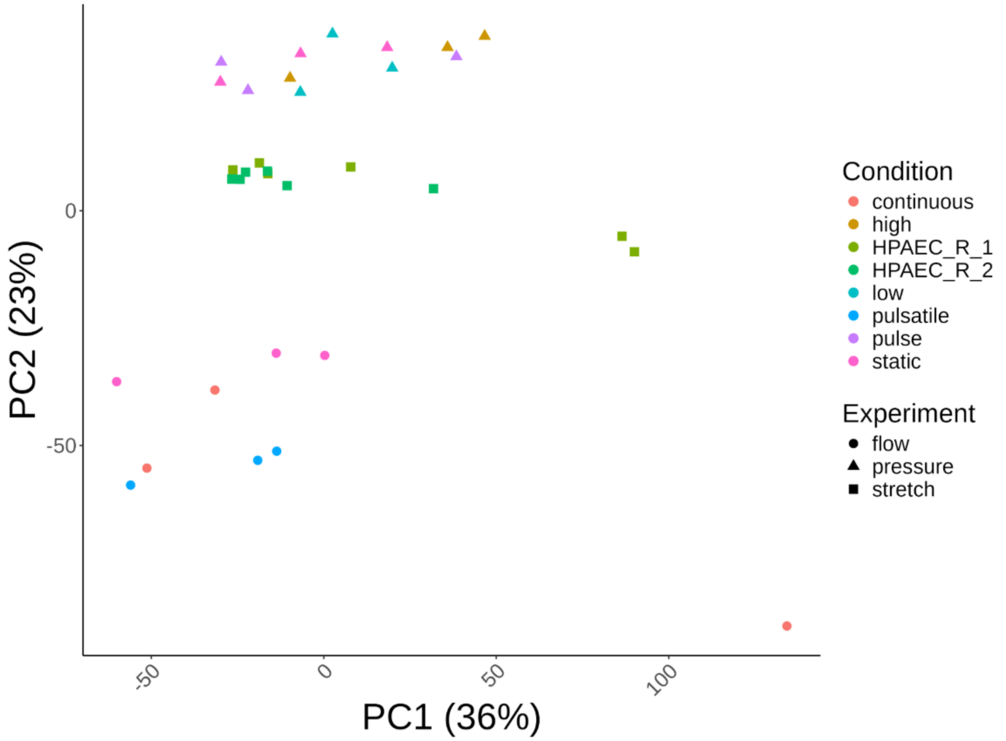

Supplemental Figure 5: PCA plots of bulk RNAseq data. Principal component analysis of the bulk RNA sequencing data used in the analysis. Percentages on axes denote percent of variation accounted for by each principal component. HPAEC_R_1 and _2 denotes no stretch and pulsatile stretch conditions, respectively.

Supplemental Fig 6: Human lung cell-specific expression of stretch- induced genes

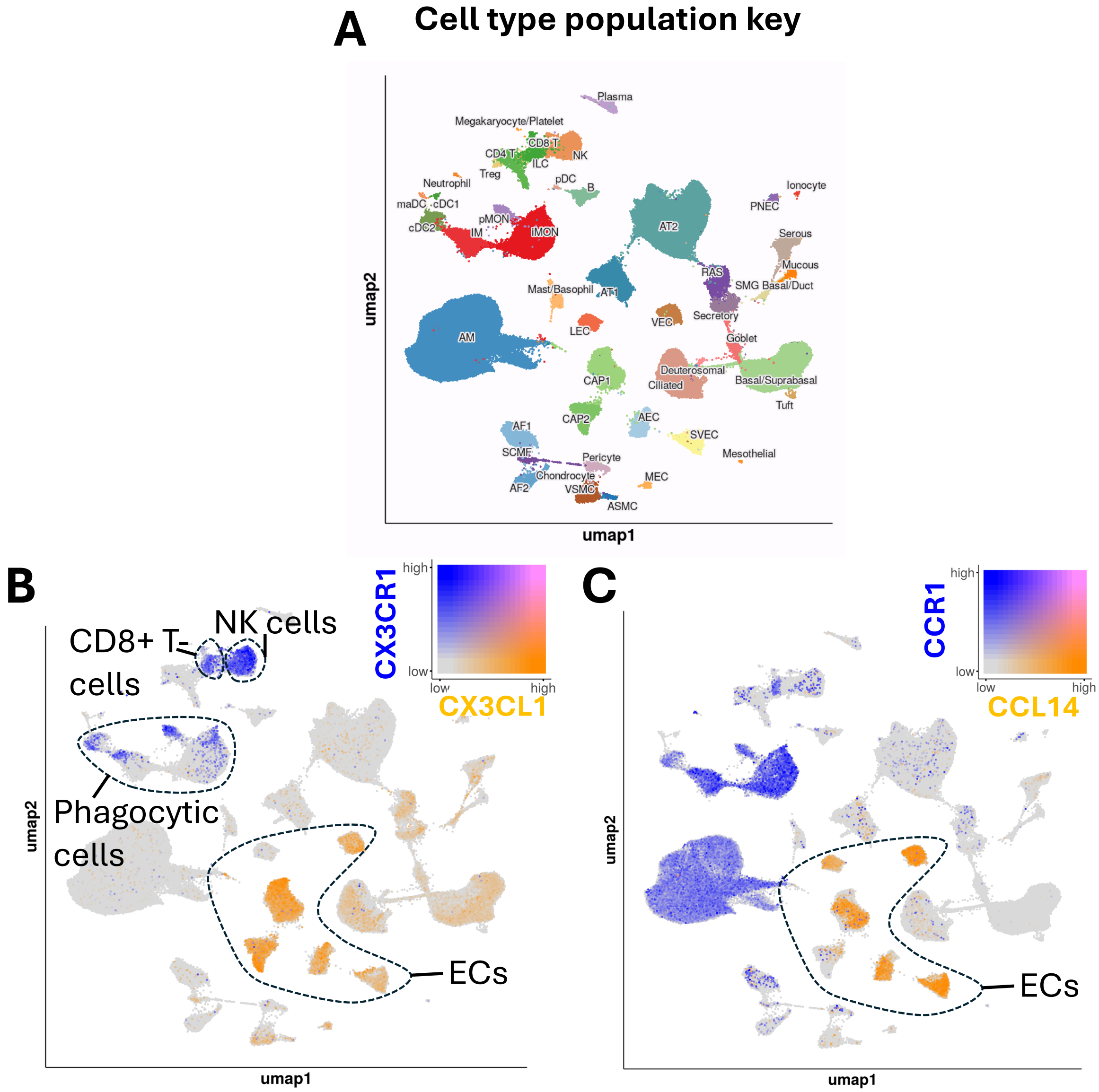

Supplemental Fig 6: Human lung cell-specific expression of stretch-induced genes. (A) LungMAP single-cell RNAseq atlas of normal human lung tissue with cell clusters identified. **(B)** CXCL3 is highly expressed by ECs, except lymphatic endothelial cells, and receptors are found in various immune cell populations. **(C)** CCL14, induced by pulsatile flow, is highly expressed by CAP1 and fewer CAP2 cells. AEC, arterial endothelial cells; SVEC, systemic vein endothelial cells; VEC, pulmonary venous endothelial cells; LEC, lymphatic endothelial cells. Full list of abbreviations in (A) can be found on www.lungmap.net.

Supplementary Figure 6: Diagram of rat Glenn surgery

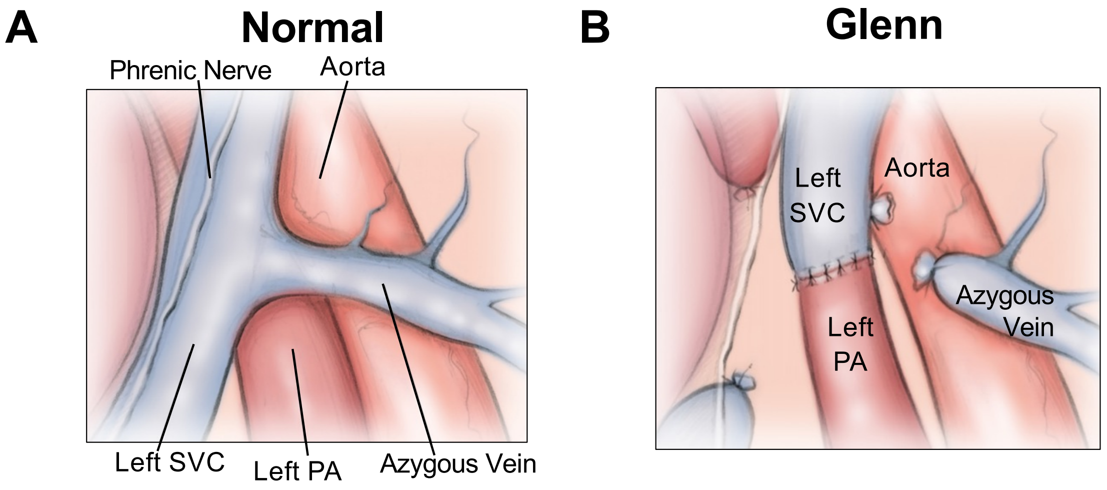

Supplementary Figure 7: Diagram of rat Glenn surgery. (A) Schematic of normal rat vascular anatomy and (**B**) rat Glenn surgery (adapted from Wan 2024 with permission).

Supplemental Table 1: Patient Demographics

	Glenn	Controls
Demographics	n = 20	n = 17
Age in years	4.4 (1.5)	4.5 (0.9)
Male	12 (60%)	8 (90%)
Cardiac Diagnosis		
Single ventricle - Glenn stage	20 (100%)	
Systemic left ventricle	9 (45%)	17 (100%)
Heart Transplantation		9
Atrial septal defect		4
Coarctation of the aorta		2
Bicuspid aortic valve		1
Ebsteins Anomaly		1
Hemodynamic State		
Cardiac Index	4.1 (0.5)	2.8 (0.5)

Supplemental Table 1: Patient Demographics. Descriptive statistics of patient data included in the study. Data is presented as mean (standard deviation) unless otherwise noted.

Supplemental Table 2: Comparison of Pulsatility Index and Pulse Difference

max	min	mean	PD	PI
20	5	10	**15**	**1.5**
30	5	16.2	**25**	**1.5**
40	10	20	**30**	**1.5**
50	15	23	**35**	**1.5**

Supplemental Table 2: Comparison of Pulsatility Index and Pulse Difference. Comparison of pulsatility index (PI) and pulse difference (PD) shows that PI can be unchanged for a variety of different PD.
